# Frauds on German Soldiers

**Published:** 1915-08-14

**Authors:** 


### FRAUDS ON GERMAN SOLDIERS.

In practically all countries since the war began there has arisen a great demand for articles in a compact form suitable to send to soldiers as tokens of affection. Often these presents take the form of foodstuffs, and those who have noticed the advertisements in daily papers and magazines have no doubt observed that occasionally even the proprietor of one patent medicine or another has endeavoured to share in the profits accruing from the generous acts of the public by drawing attention to the value of a particular concoction as an adjunct to trench life. But, fortunately, no general attempt seems to have been made in this country to take advantage of public generosity by advertising worthless "fakes."

But this restraint has not been universal, for both in France and Germany attention has been directed to the existence of a most dishonourable traffic in faked foods and other articles. According to a report by the Director of the Chemical Laboratory of the Department of Police in Berlin, particulars of which have reached this country through the medium of the official organ of the American Medical Association, this disgraceful traffic has developed some unique novelties in the way of fraudulent food products. One of these is described as "solid alcohol" and is advertised as a substitute for familiar spirituous beverages. It consists of cubes of gelatin to which brandy and sugar are supposed to have been added before the mixture has solidified. The soldier is directed to pour hot water on the cubes in order to produce a glass of "punch." The Berlin police chemists who have examined this liquid find that it contains practically no alcohol and possesses the disagreeable flavour of glue. Other so-called substitutes for alcoholic beverages which the relatives of soldiers are induced by flattering advertisements to send to the Front are often nothing more than cubes of sugar coloured red with aniline dye and flavoured with tartaric acid. It is possible that the soldiers are better without alcohol, but it is from no altruistic motive that the manufacturers of these fraudulent compounds have been carrying on such a profitable trade. Coffee and cocoa have also been sold in tablet form in large quantities. The Berlin police report that these are often of the most inferior quality and sold at exorbitant prices. One brand of coffee tablets which had become very popular before the police took action sold at the rate of twelve shillings a pound; the packets appear to have been wrapped in attractive covers bearing ennobling messages to the heroes of the war! Tablets supposed to be composed of dried milk have also been sent in enormous quantities to the German soldiers; some brands of these have been reported as very inferior. The food faker, never a patriot whatever his nationality, is intolerable in time of peace, but in time of war he is a heartless villain.

